# Supplementary material for: CDC42-IQGAP Interactions Scrutinized: New Insights into the Binding Properties of the GAP-Related Domain
Source: Int J Mol Sci. 2022 Aug 9;23(16):8842. doi: 10.3390/ijms23168842 (PMC9408373; doi:10.3390/ijms23168842)
Supplement: Supplementary file 1 [file ijms-23-08842-s001.zip › ijms-1808071-supplementary.pdf]

CDC42-IQGAP interactions scrutinized: new insights into the binding properties of the GAP-related domain

Niloufar Mosaddeghzadeh, Silke Pudewell, Farhad Bazgir, Neda S Kazemein Jasemi, Oliver HF Krumbach, Lothar Gremer, Dieter Willbold, Radovan Dvorsky, Mohammad R. Ahmadian

Institute of Biochemistry and Molecular Biology II, Medical Faculty and University Hospital Düsseldorf, Heinrich Heine University Düsseldorf, 40225 Düsseldorf, Germany

Table S1. List of CDC42 and IQGAP variants.

	Variant names	Details
CDC42	$CDC42^{WT}$	CDC42 wild type
	$CDC42^{2xSW}$	CDC42 with 2 mutations in the switch regions I and II (P34A, Y64A)
	$CDC42^{8xSW}$	CDC42 with 8 mutations in the switch regions I and II (P34A, V36A, F37A, D38A, D63A, Y64A, R66A, L67A)
	$CDC42^{S124D}$	CDC42 with mutation in insert helix S124D
	$CDC42^{A130K}$	CDC42 with mutation in insert helix A130K
	$CDC42^{K131E}$	CDC42 with mutation in insert helix K131E
	$CDC42^{N132K}$	CDC42 with mutation in insert helix N132K
	$CDC42^{4xIH}$	CDC42 with 4 mutations in insert helix (S124D, A130K, K131E, N132K)
IQGAP1	$C794^{WT}$	aa 863-1657
	$GRD1^{WT}$	aa 962-1345
	$GRD1^{T1046R}$	aa 962-1345 with T1046R mutation in “arginine” finger
	CT	aa 1567-1657
IQGAP2	$C795^{WT}$	aa 780-1575
	$C795^{11xGRD}$	C795 with 11 mutations within the proposed CDC42 binding regions (Y1106A, R1107A, N1110A, P1111A, V1114G, D1117A, G1118A, F1119A, S1132A, R1135A, R1136A)
	$GRD2^{WT}$	aa 875-1246
IQGAP3	$GRD3^{WT}$	aa 942-1330

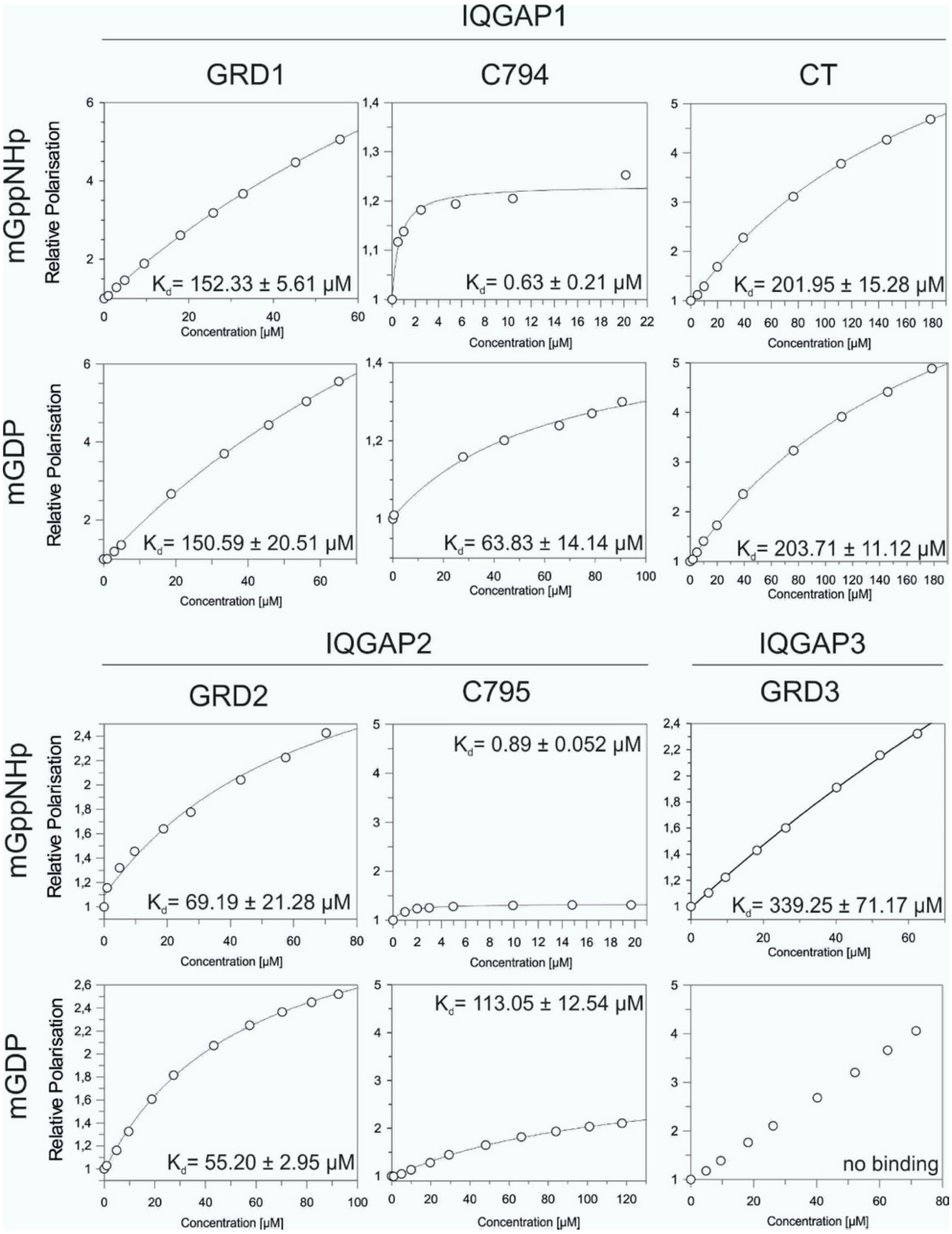

Figure S1. Fluorescence polarization measurements for binding of IQGAP 1/2/3 constructs to CDC42 WT. CDC42 WT in mant-GppNHp or mant-GDP state was titrated with increasing amounts of IQGAP1 GRD, C794 and CT; IQGAP2 GRD2 and C795 and IQGAP3 GRD and fluorescence polarisation were measured. Determined K_d values are displayed in the graphs in part illustrated as bar charts in Figure 1B.

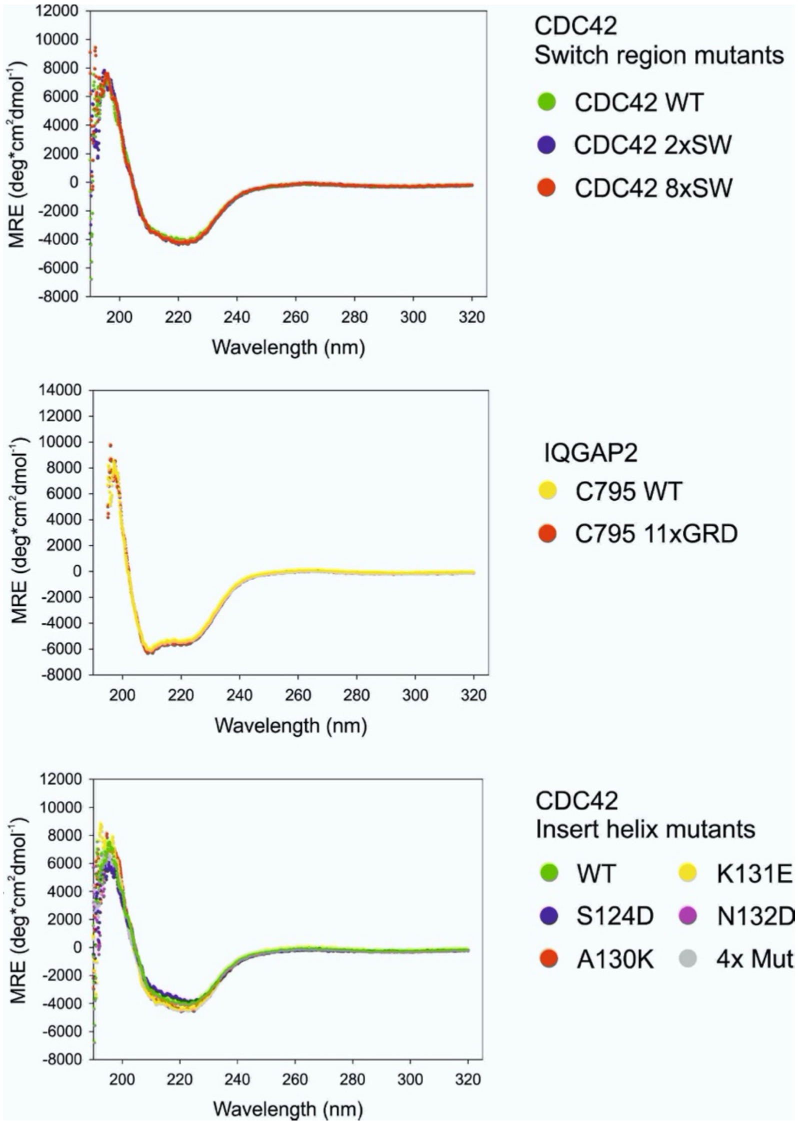

Figure S2. Circular dichroism spectrometry measurements of CDC42 WT and all mutations in CDC42 switch region and insert helix and IQGAP2 C795 WT and C795 11xGRD. CDC42 switch region constructs with 2- and 8-mutations, the insert helix mutants S124D, A130K, K131W, N132D, and the 4-residues mutant as well as the IQGAP2 constructs C795 WT and C795 11xGRD were analysed for folding by CD measurements. No folding changes could be observed for any of the proteins compared to the wild type.

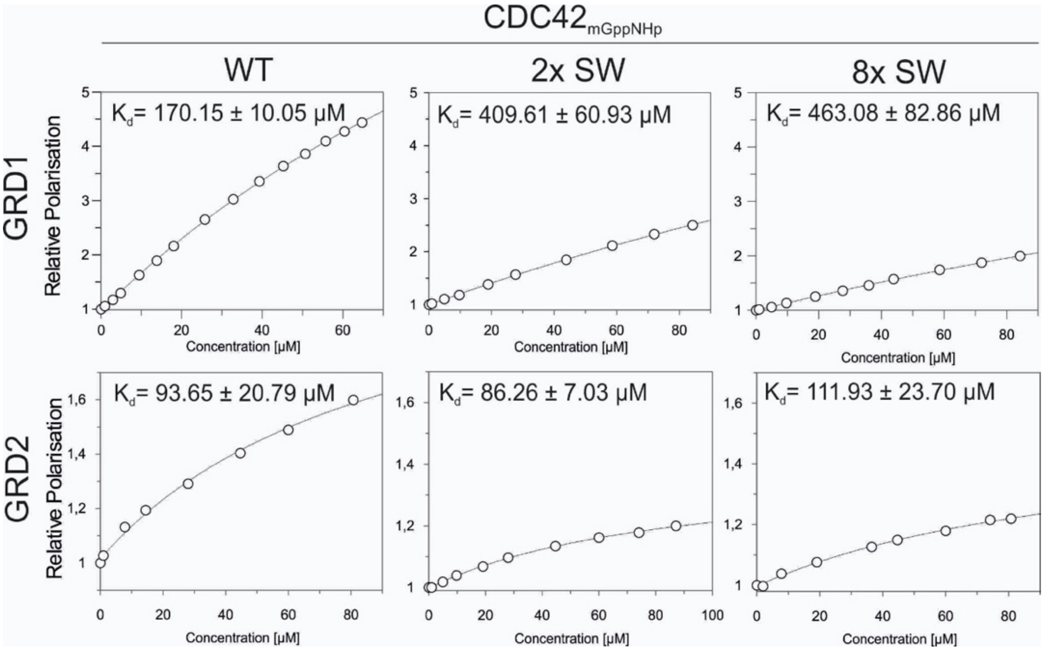

Figure S3. Fluorescence polarization measurements for binding of CDC42 WT and SW mutants to IQGAP GRD1. CDC42 WT, the 2x and 8x mutant in swich region was mant-GppNHp labelled and measured with increasing concentrations of IQGAP GRD1. K_d values are displayed in the graphs. Determined K_d values are displayed in the graphs in part illustrated as bar charts in Figure 2C.

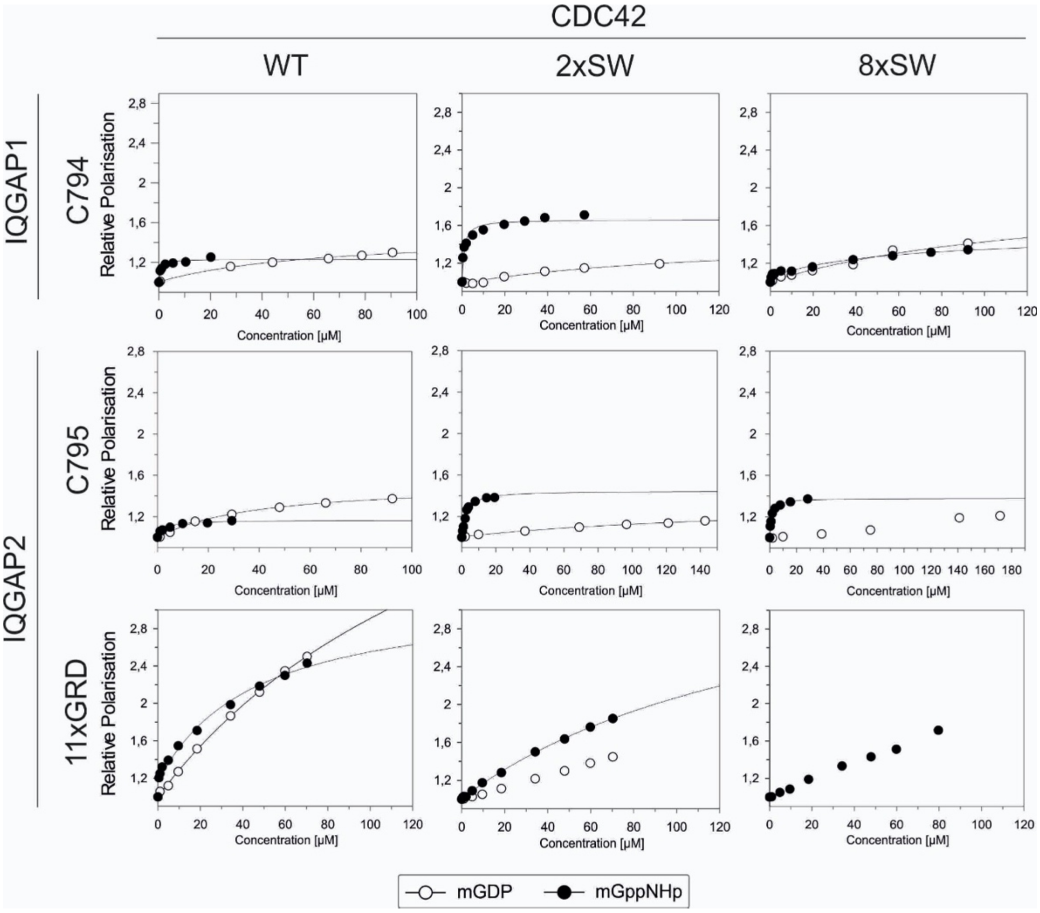

Figure S4. Fluorescence polarization measurements for binding of CDC42 constructs to IQGAP C795 constructs. CDC42 WT, 2x and 8x mutants in switch I and II region, either in mant-GDP (white points) or mant-GppNHp (black points) state were used, titrated with increasing amounts of IQGAP2-C795 WT, 11x or IQGAP1-C794 and fluorescence polarisation changes were recorded. K_d values of fitted data in µM are displayed in the table below. Datapoints without a line can not be fitted with applied non-linear fits. The C794 and C795 WT measurements with CDC42 mGppNHp and mGDP are analyzed in Figure S1. Determined K_d values are illustrated as bar charts in Figure 2D.

	CDC42 WT		CDC42 2xSW		CDC42 8xSW	
	mGppNHp	mGDP	mGppNHp	mGDP	mGppNHp	mGDP
IQGAP1 C794	0,63 ± 0,21	63,83 ± 14,14	1,04 ± 0,29	123,57 ± 56,76	54,01 ± 23,83	139,77 ± 53,76
IQGAP2 C795	0,89 ± 0,052	113,05 ± 12,54	2,38 ± 0,40	193,70 ± 41,89	1,34 ± 0,13	No binding
IQGAP2 C795 11xGRD	45,55 ± 14,84	173,30 ± 24,96	162,21 ± 20,51	No binding	No binding	Not measured

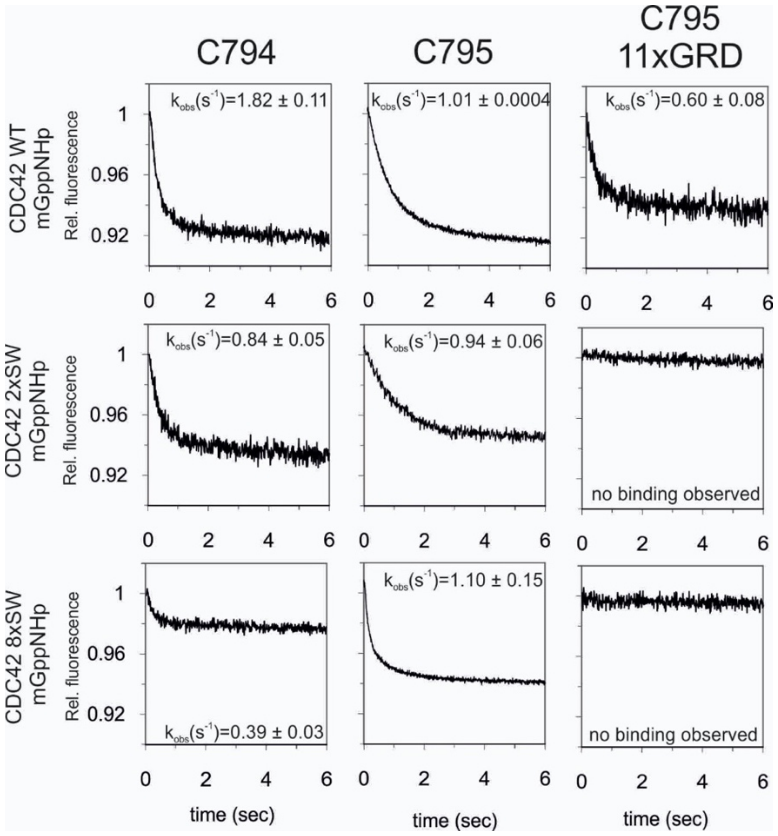

Figure S5. Stopped-Flow measurements for binding of CDC42 WT and mutants to IQGAP1 and IQGAP2 constructs. CDC42 WT, 2xSW and 8xSW mutants in switch I and II region, either in mant-GDP or mant-GppNHp state were measured with IQGAP1 C794 and IQGAP2 C795 and C795 11xGRD mutant. Measured data are normalized to 1 and k_{obs} values are determined by fitting the datapoints. Determined k_{obs} values are illustrated as bar charts in Figure 2E.

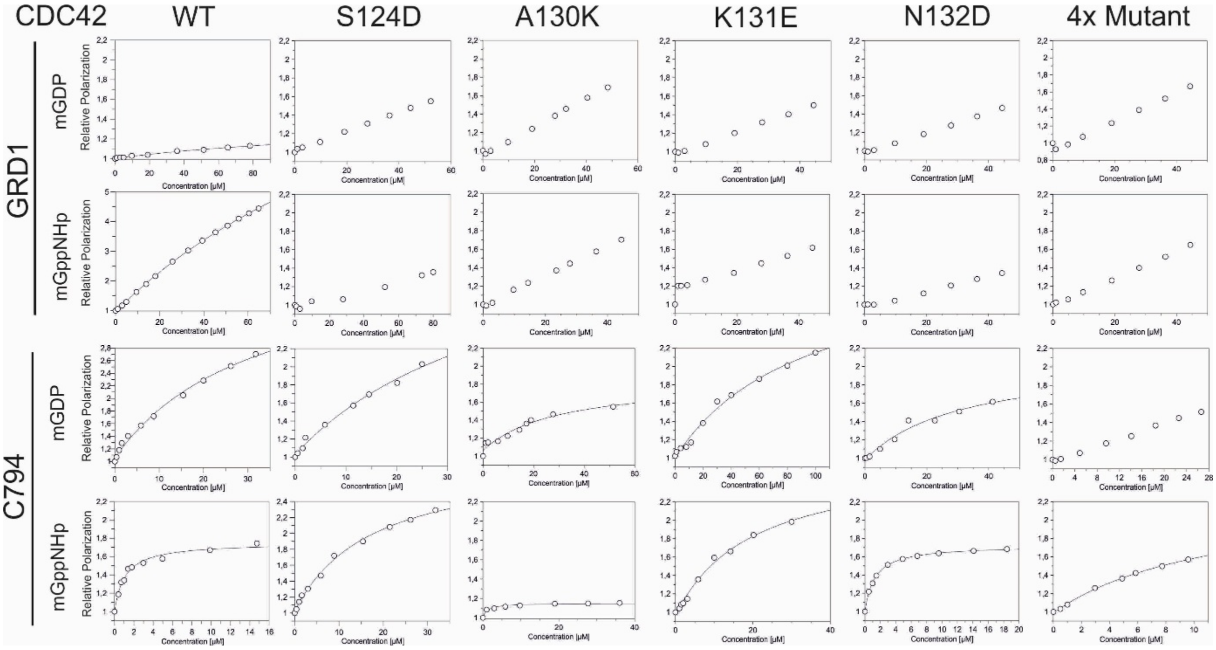

Figure S6. Fluorescence polarization measurements for binding of CDC42 WT or mutantsto IQGAP1 GRD1 or C794. CDC42 WT and insert helix mutations S124D, A130K, K131E, N132D and the 4x mutant are either in mant-GDP or mant-GppNHp loaded state. K_d values of fitted data are displayed in the supplementary table below. The data for GRD1 binding to CDC42 WT mGppNHp is shown in figure S3. Determined K_d values are illustrated as bar charts in Figure 3B.

		IQGAP1 GRD1	IQGAP1 C794
CDC42 WT	mGppNHP	170,15 ± 10,05	1,07 ± 0,17
	mGDP	186,93 ± 77,45	32,69 ± 6,93
CDC42 S124D	mGppNHP	No binding	17,46 ± 2,30
	mGDP	No binding	44,45 ± 13,04
CDC42 A130K	mGppNHP	No binding	1,21 ± 0,37
	mGDP	No binding	29,18 ± 13,22

		IQGAP1 GRD1	IQGAP1 C794
CDC42 K131E	mGppNHP	No binding	19,75 ± 3,92
	mGDP	No binding	93,65 ± 25,02
CDC42 N132K	mGppNHP	No binding	1,27 ± 0,05
	mGDP	No binding	31,24 ± 13,94
CDC42 4xIH	mGppNHP	No binding	11,92 ± 1,87
	mGDP	No binding	No binding

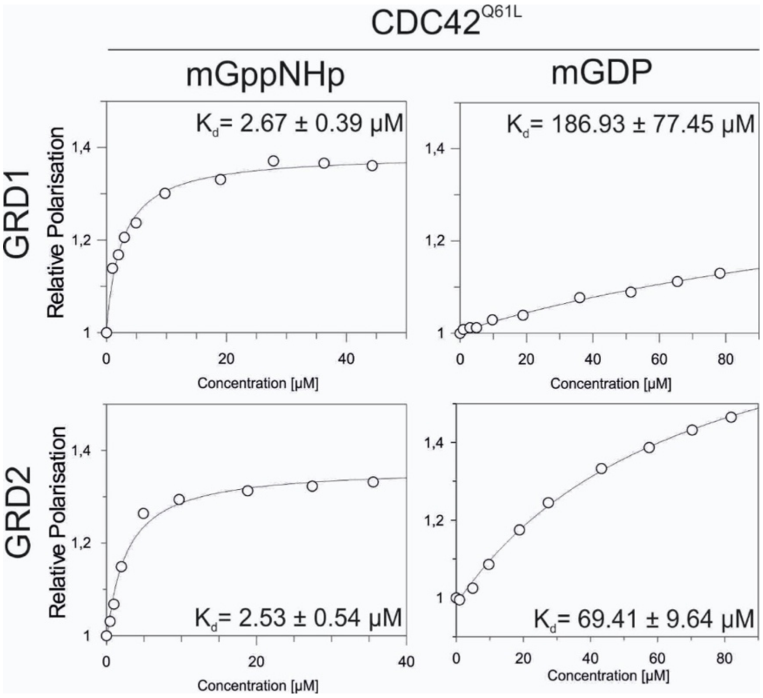

Figure S7. Fluorescence polarisation measurements for binding of CDC42 Q61L to IQGAP GRD1 or IQGAP GRD2. The CDC42 Q61L mutant was labelled with mant-GppNHp or mant-GDP,titrated with the GRD of IQGAP1 or IQGAP2 and fluorescence polarisation changes were determined. Determined K_d values are illustrated as bar charts in Figure 4B.
